# Altered Functional Response to Risky Choice in HIV Infection

**DOI:** 10.1371/journal.pone.0111583

**Published:** 2014-10-27

**Authors:** Colm G. Connolly, Amanda Bischoff-Grethe, Stephan J. Jordan, Steven Paul Woods, Ronald J. Ellis, Martin P. Paulus, Igor Grant, for The Translational Methamphetamine AIDS Research Center (TMARC) Group

**Affiliations:** 1 Dept of Psychiatry, University of California San Francisco, San Francisco, California, United States of America; 2 Dept of Psychiatry, University of California San Diego, La Jolla, California, United States of America; 3 Psychiatry Service, VA San Diego Healthcare System, La Jolla, California, United States of America; 4 HIV Neurobehavioral Research Program, University of California San Diego, San Diego, California, United States of America; 5 Department of Neurosciences, University of California San Diego, San Diego, California, United States of America; UCL Institute of Child Health, University College London, United Kingdom

## Abstract

**Background:**

Risky decision-making is commonly observed in persons at risk for and infected with HIV and is associated with executive dysfunction. Yet it is currently unknown whether HIV alters brain processing of risk-taking decision-making.

**Methods:**

This study examined the neural substrate of a risky decision-making task in 21 HIV seropositive (HIV+) and 19 seronegative (HIV-) comparison participants. Functional magnetic resonance imaging was conducted while participants performed the risky-gains task, which involves choosing among safe (20 cents) and risky (40/80 cent win or loss) choices. Linear mixed effects analyses examining group and decision type were conducted. Robust regressions were performed to examine the relationship between nadir CD4 count and Kalichman sexual compulsivity and brain activation in the HIV+ group. The overlap between the task effects and robust regressions was explored.

**Results:**

Although there were no serostatus effects in behavioral performance on the risky-gains task, HIV+ individuals exhibited greater activation for risky choices in the basal ganglia, i.e. the caudate nucleus, but also in the anterior cingulate, dorsolateral prefrontal cortex, and insula relative to the HIV- group. The HIV+ group also demonstrated reduced functional responses to safe choices in the anterior cingulate and dorsolateral prefrontal cortex relative to the HIV- group. HIV+ individuals with higher nadir CD4 count and greater sexual compulsivity displayed lower differential responses to safe versus risky choices in many of these regions.

**Conclusions:**

This study demonstrated fronto-striatal loop dysfunction associated with HIV infection during risky decision-making. Combined with similar between-group task behavior, this suggests an adaptive functional response in regions critical to reward and behavioral control in the HIV+ group. HIV-infected individuals with higher CD4 nadirs demonstrated activation patterns more similar to seronegative individuals. This suggests that the severity of past immunosuppression (CD4 nadir) may exert a legacy effect on processing of risky choices in the HIV-infected brain.

## Introduction

The human immunodeficiency virus (HIV) can cross the blood-brain barrier early in the course of infection and trigger a cascade of functional and structural alterations [1], [2]. One of the primary loci of this damage is the deep grey matter of the basal ganglia [2] which are reciprocally connected to a broad range of cortical regions [3]–[5]. HIV infection has been reliably linked to injury of the fronto-striato-thalamo-cortical loops [6], and can thus adversely impact a variety of higher-order neurocognitive functions that rely on these circuits. For example, HIV-associated neurocognitive deficits have been observed in approximately half of infected persons in such ability areas as fine-motor skills [2], working memory [7], and executive function [8].

Risk-taking and reward processing are important processes that influence behavior [9]. Making a choice in a risky situation typically requires a choice between an option that is associated with a large outcome that may be either advantageous or disadvantageous versus an alternative with a smaller, more certain advantageous outcome [10], [11]. One formulation of risk is its econometric definition as the variance of the value of the possible outcomes [12]. This conception, however, does not account for the influences of emotion since human decision-making is not always rational [13]. More recent approaches attempt to bridge the gap between rational choice and emotions guiding decisions. For example, the somatic marker hypothesis [14], [15], affect heuristic hypothesis [16] and risk-as-feelings hypothesis [17] posit that emotions are integrated with cognitive evaluation of choices to regulate the decision making process.

Risky decision-making is commonly observed in individuals at risk for (e.g., [18]) and infected with (e.g., [19], [20]) HIV. For example, on the Iowa Gambling Task (IGT), HIV-infected (HIV+) individuals made disproportionately more selections from “bad” decks as the task progresses (e.g., [19], [20]), which may reflect poor inhibition response to the lure of high rewards even in the face of large penalties [20]. Such risky decision-making is more common in individuals with HIV-associated Neurocognitive Disorders (HAND; [21]) and has been specifically, but not consistently, linked to cognitive inflexibility and engagement in HIV transmission risk behaviors (e.g., [22]). Additionally, personality characteristics such as sensation seeking, that is a propensity to seek out novel, exciting and arousing stimuli, have been associated with risk behaviors for HIV infection [23], [24]. However, few studies have examined the neural substrates of risky decision-making in HIV.

A number of neuroimaging studies have shown that HIV infection can alter brain function [25]–[33]. In functional magnetic resonance imaging (fMRI) experiments, HIV+ individuals have been shown to exhibit deficits on tests of attention and working memory and altered responses within the associated neural substrates [25]–[27], [31], [32]. In these tasks, HIV+ individuals exhibited greater activation and/or larger load dependent increases in the frontal and parietal cortical regions underlying task performance [25]–[27], [31], a pattern that is consistent with damage to fronto-striatal white matter tracts that has been attributed to HIV infection [34]. Preliminary evidence suggests that hippocampal function can be affected by HIV infection [28]. Reduced hippocampal activation during encoding and elevated hippocampal activation during the recall phase of a verbal memory task having been reported in a sample of HIV+ women [28]. There has been a recent interest in the use of magnetoencephalography (MEG) in the study of HIV infection in part due to its high test-retest reliability in HIV infection [35]. These studies have reported strong responses (in the 8–13 Hz band) in the dorsolateral prefrontal cortex (DLPFC) during a simple finger tapping task [30]. Reduced levels of synchronization (in the 6–12 Hz band), recorded during a simple visual processing task, have been also been observed in a HIV+ compared to a control group [29]. Furthermore, those HIV+ individual with desynchronization more similar to controls demonstrated better performance on a neuropsychological test of verbal learning [29]. Notably, cortical thinning in the DLPFC has been linked to the severity of immune suppression in HIV infection [36]. Finally, reduced functional connectivity has been reported in resting state MEG [37] and in a fMRI task studying semantic event sequencing [38]. Together, neuroimaging studies showing preferential involvement of fronto-basal ganglia brain systems in HIV-infection [25], [33], [38] and neuropsychological studies of the maladaptive use of feedback in risky choice [8], [19], [20], [39] lay the foundation for fMRI studies to more directly link impaired behavior with abnormalities of the brain substrates of risky decision-making and reward in HIV infection.

Extensive study of risk-taking in the context of decision-making has revealed a key network of cortical and subcortical brain regions. This network is composed of circuits, two of which, namely the ventral limbic circuit and the dorsal executive circuit, may be important to choice behavior. The ventral circuit includes several striatal structures: the nucleus accumbens, rostromedial caudate, rostroventral putamen, and ventromedial caudate [40]. These regions receive extensive innervation from prefrontal cortical regions such as the orbitofrontal and ventromedial cortex [41], [42] and insula [40]. This circuit is implicated in the identification of rewarding and emotionally salient stimuli, integrating these with autonomic, visceral, and hedonic information, and generating affective responses to these stimuli [43]–[48]. The dorsal executive circuit encompasses the dorsal caudate, dorsal anterior cingulate cortex (DACC), and DLPFC. In this circuit DACC is thought to play a role in performance monitoring [49]–[53] whereas DLPFC is thought to be important to the maintenance of goal-directed behavior [54], [55]. Indeed, in the context of risky-choice behavior, modulation of activity within the DLPFC can lead to different response styles under risk [56]–[58]. The anterior cingulate cortex (ACC) and DLPFC are extensively interconnected [59] with the DACC also projecting to the dorsal caudate [40]. This circuit is important to selective attention, planning and effortful regulation of affective states, including task switching and inhibition. Acting in concert, these two circuits may code stimulus-reward value, maintain representations of predicted future reward and future behavioral choice, and transform decisions into motor output, playing a role in integrating and evaluating reward prediction to guide decisions.

This study aimed to examine whether HIV alters brain processes underlying risk-taking decision-making. We hypothesized that greater activity for risky choices would be observed in grey matter basal ganglia regions. Additionally, we theorized that dorsolateral and anterior cingulate cortex would display greater activity in the HIV+ group relative to HIV- comparisons, and that this may be an adaptive functional response to compensate for aberrant information provided by other cortical and subcortical structures that may have been impacted by HIV infection. Finally, we explored whether nadir cluster of differentiation 4 (CD4) count, a measure of historical immune function that has been shown to predict neurocognitive impairment [60]–[64] and structural volumes [65] in HIV+ individuals, would demonstrate any association with functional brain measures of risky decision-making.

## Materials and Methods

### Ethics Statement

The University of California, San Diego human research protection program approved this study. Participants gave informed written consent and were compensated for their time and effort.

### Participants

Participants were recruited as part of the Translational Methamphetamine AIDS Research Center and included 21 HIV+ and 19 seronegative comparison adults. HIV status was confirmed by MedMira Multiplo rapid test (MedMira Inc., Nova Scotia, Canada). All participants were seronegative for Hepatitis C virus (HCV) as determined by the MedMira Multiplo rapid test. Current CD4 T lymphocyte counts (cells/ml) were determined by flow cytometry at a medical center laboratory certified by Clinical Laboratory Improvement Amendments (CLIA), or CLIA equivalent. HIV RNA levels were measured in plasma by reverse transcriptase PCR (Roche Amplicor, v. 1.5, lower limit of quantitation 50 copies/ml). CD4 nadir was obtained by self-report, with confirmation by documented prior measurements in a subset of individuals. Participants were excluded if they tested positive for illicit drugs (with the exception of marijuana (MJ)) or alcohol (urine toxicology screen or Breathalyzer respectively) on the day of scan; had contra-indications for MRI; had a lifetime history of schizophrenia or other primary psychotic disorders; had previous cerebrovascular events, determined by comprehensive neurological exam; head injury with loss of consciousness for greater than 30 minutes or resulting in neurologic complications; seizure disorder; demyelinating diseases from non-HIV neurological disorders; met Diagnostic and Statistical Manual of Mental Disorders (4^th^ Edition-Text Revision; DSM-IV-TR) [66] conditions for substance (other than alcohol, MJ and nicotine) abuse in the prior year or dependence within the preceding five years. Participants who met criteria for lifetime dependence or abuse of MJ within the last 12 months were enrolled. Those who met lifetime criteria for alcohol abuse within the prior 12 months were enrolled but were excluded if they met criteria for dependence within the previous 12 months. Nicotine use was not exclusionary and participants were told not to alter their typical pattern of daily usage. They were asked to refrain from smoking during the break in the scanning session. Breath carbon monoxide levels and the presence of cotinine in urine were assessed on the day of the scan.

### Participant Assessment

Assessment took place on two separate visits: an initial neurological and neuropsychiatric visit and a subsequent MRI scan (see Table 1 for the interval between visits). Determination of relevant psychiatric diagnoses were achieved using the Composite International Diagnostic Interview (CIDI 2.0) [67], a computer based structured interview administered by a trained research associate on the initial visit. This evaluation tool yields lifetime and current (within 1 month) diagnoses that are consistent with DSM-IV-TR [66] criteria. The resultant measures of mood and substance use disorders were used to inform eligibility for study enrollment and characterizing the sample. Additionally, participants were asked their age, gender, handedness, and sexual orientation.

**Table 1:** Characteristics of the HIV- and HIV+ groups.

Characteristic	HIV- a	HIV+ a	DoF	Statistic b	P-value	Effect Size c	Significance
**Demographic Characteristics**							
Number of Participants	19	21	1.00	χ2 = 0.03	0.87		
Gender (M/F)	18/1	19/2	1.00	χ2≈0	1.00		
Sexual Orientation (MSM/Heterosexual)	5/14	18/3	1.00	χ2 = 12.07	<0.001		***
Handedness (R/L)	18/1	18/3	1.00	χ2 = 0.18	0.67		
Age at time of scanning (years)	38.1±2.5 (23–54)	40.8±2.6 (23–58)	37.99	t = −0.74	0.46		
Years of education	14.4±0.5 (11–20)	13.8±0.5 (9–18)	37.97	t = 0.85	0.40		
Days between initial and scanning visits†	58±37.1 (19–124)	77±44.5 (23–733)	NA	W = 135.5	0.09	g = 0.34	
**Ethnicity (%)**			3.00	χ2 = 3.85	0.28		
African-American	2.5	12.5					
Hispanic	12.5	10					
Other	0	2.5					
White	32.5	27.5					
**Scanner (n)**			1.00	χ2 = 5.05	0.02		
East	10	3					
West	9	18					
**Dates of scan acquisition** d							
East	2011/02/03–2011/08/10	2010/06/05–2010/08/14					
West	2011/04/01–2012/04/04	2011/01/03–2012/03/19					
**Psychiatric Characteristics**							
Wide Range Achievement Test	106.1±2.7 (94–134)	99.7±2.2 (85–134)	35.81	t = 1.85	0.07	g = 0.60	
Global Deficit Score†	0.2±0.2 (0–1)	0.3±0.3 (0–1.9)	NA	W = 151.5	0.20	PS = 0.38	
Global Deficit Score Impaired (n)	3	7	1.00	W = 0.84	0.36		
Speeded Information Processing	51.8±1.9 (38.8–69.5)	48.1±1.9 (31.5–63.8)	37.95	t = 1.38	0.17	g = 0.40	
Verbal Fluency	49.2±1.9 (38–66.7)	46.5±1.6 (35.3–64.7)	35.92	t = 1.10	0.28	g = 0.30	
Learning	46.8±2.2 (20–58)	42.3±1.6 (24.5–56)	32.91	t = 1.63	0.11	g = 0.50	
Working Memory	51.7±2.3 (28.5–68)	47±1.5 (33.5–59.5)	31.28	t = 1.68	0.10	g = 0.50	
Executive Functions	51.1±2.2 (34.5–67.8)	46±2 (29.2–68.5)	37.07	t = 1.7	0.10	g = 0.50	
Motor Skills	52.4±2.4 (38–71.5)	49.7±2.5 (33.5–72)	38.00	t = 0.78	0.44	g = 0.20	
BDI-II (at initial visit)	1.7±0.6 (0–7)	10.9±2.2 (0–35)	22.53	t = −4.07	0.00	g = −1.3	***
BDI-II (at time of scan)	1.7±0.8 (0–14)	8.6±2 (0–35)	25.71	t = −3.16	<0.01	g = −1.00	**
POMS (at time of scan)	55.8±3.3 (25–78)	68.8±6.6 (27–146)	29.02	t = −1.75	0.09	g = −0.50	
Lifetime MDD Diagnosis (n)	3	11	1.00	χ2 = 4.37	0.04		*
Current MDD Diagnosis (n)	0	5	1.00	χ2 = 3.22	0.07		
**Kalichman Sexual Sensation Seeking Scale**							
Sexual Sensation Seeking Scaled (Mean)	1.8±0.1 (1.2–2.6)	2.1±0.1 (1–3.3)	34.04	t = −1.79	0.08	g = −0.60	
Non Sexual Sensation Seeking Scaled (Mean)	2.3±0.1 (1.5–3.5)	2±0.1 (1.3–3)	36.32	t = 2.02	0.05	g = −0.60	*
Sexual Compulsivity Scaled (Mean)	1.1±0 (1–1.6)	1.4±0.1 (1–2.4)	26.01	t = −2.33	0.03	g = −0.70	*
**Barratt Impulsiveness Scale**							
Total	59.3±2.3 (46–79)	61.1±2.4 (45–85)	38.00	t = −0.54	0.59		
Attentional Subscale	15.3±0.7 (9–20)	16.6±0.8 (9–22)	37.86	t = −1.16	0.25		
Motor Subscale	18.1±0.8 (13–30)	19.1±0.9 (13–27)	37.96	t = −0.84	0.41		
Non Planning Subscale	26±1.2 (16–38)	25.5±1.1 (16–38)	37.05	t = 0.32	0.75		
**Clinical Characteristics**							
Duration of Infection (months)	Not applicable	96.9±22.8 (1.6–318.1)					
Nadir CD4 Count†	Not applicable	250±174.9 (3–763)					
Current CD4 Count†	Not applicable	426±277.2 (81–1061)					
Plasma Viral Load (% Detectable)	Not applicable	42.90					
AIDS (%)	Not applicable	47.60					
ART Currently Prescribed (%)	Not applicable	71.43					
ART Past Use (%)	Not applicable	9.52					
ART Never Used (%)	Not applicable	19.05					
**Current Substance Use Characteristics**							
Fagerström Test for NicotineDependence (Total Score)	1.6±0.6 (0–8)	1.8±0.6 (0–9)	38	t = −0.22	0.82		
Nicotine Dependence (%)	0	5 (23.8)	1	χ2 = 3.22	0.07		
Alcohol Abuse (%)	1 (5.3)	0	1	χ2≈0	0.96		
Alcohol Dependence	0	0					
Cannabis Abuse	0	0					
Cannabis Dependence	0	0					
**Lifetime Substance Use Characteristics**							
Alcohol Abuse (%)	5 (26.3)	5 (23.8)	1	χ2≈0	1		
Alcohol Dependence (%)	2 (10.5)	1 (4.8)	1	χ2 = 0.01	0.93		
Cannabis Abuse (%)	5 (26.3)	4 (19.0)	1	χ2 = 0.03	0.86		
Cannabis Dependence (%)	0	1 (4.8)	1	χ2≈0	1		
Cocaine Abuse	0	0					
Cocaine Dependence	0	0					
Methamphetamine Abuse	0	0					
Methamphetamine Dependence	0	0					
Hallucinogen Abuse (%)	1 (5.3)	0	1	χ2≈0	0.96		
Hallucinogen Dependence (%)	0	1 (4.8)	1	χ2≈0	1		
Inhalant Abuse	0	0					
Inhalant Dependence	0	0					
Opioid Abuse	0	0					
Opioid Dependence	0	0					
PCP Abuse	0	0					
PCP Dependence	0	0					
Sedative Abuse (%)	0	1 (4.8)	1	χ2≈0	1		
Sedative Dependence	0	0					
MDMA Abuse	0	0					
MDMA Dependence (%)	0	1 (4.8)	1	χ2≈0	1		

a Mean ± SEM (min - max) or median ± MAD (min - max) if indicated by †.

b Statistic: W, Wilcox rank sum test; χ2, χ2 test for equality of proportions; t, Student’s T test.

c Effect size: g, Hedge’s g; PS, probability of superiority.

d Dates are in year/month/day format.

Abbreviations: SEM, standard error of the mean; MAD, median absolute deviation; MSM, men who have sex with men; BDI-II, Beck Depression Inventory II, POMS, Profile of Mood States; MDD, Major Depressive Disorder; CD4, Cluster of Differentiation 4; AIDS, Acquired Immunodeficiency Syndrome; ART, Anti-retroviral therapy; MDMA, 3,4-methylenedioxymethylamphetamine; DoF, degrees of freedom; NA, not applicable; M, male; F, female; R, right; L, left. '*' p<0.05, '**' p<0.01.

Since HIV has been associated with cognitive impairment [68], all participants completed the oral word reading subtest of the Wide Range Achievement Test (WRAT-4) [69] on the initial visit and a comprehensive neuropsychological test battery (described in detail in [70]), performance on which was summarized using the Global Deficit Score (GDS) [68], [71], [72]. Specifically, we assessed the following domains: speeded information processing, verbal fluency, learning, working memory, executive functions, and motor skills (more details can be found in [21]). As neuropsychiatric symptoms are common in many HIV+ individuals [73], we also administered the Beck Depression Inventory-II (BDI-II) [74] and Profile of Mood State (POMS) [75] on the day of the scan. BDI-II was also measured on the initial visit. Deficits in the IGT suggest that HIV+ individuals tend to choose larger immediate rewards over smaller rewards that result in longer term gains overall [19], [20]. This suggests an impulsive response style in HIV+ individuals [19]. Consequently, we measured this using the Barratt Impulsiveness Scale-11 (BIS-11) [76]. Furthermore, since risky decision-making in HIV+ individuals may be related to sensation seeking and could represent a common pathophysiology [22], we chose to measure it using the Kalichman Sexual Sensation Seeking Scale (KS4) [24] which assesses personality characteristics and high-risk sexual behavior known to be associated with HIV transmission risk [23], [24]. Estimates of smoking rates among HIV-infected individuals are in the range of 50–70% [77], [78] with smokers being less likely to adhere to antiretroviral medication regimes [79]. We therefore assessed nicotine usage using the Fagerström Test for Nicotine Dependence (FTND) [80].

### Task

**Figure 1:**
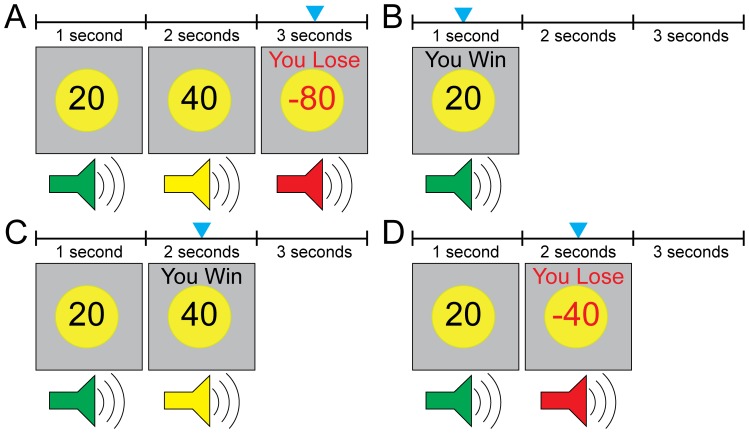
An illustration of four different trial types from the risky gains task: (A) lose 80, (B) win 20, (C) win 40 and (D) lose 40. The blue arrow indicates participant response to select the value on screen.

Participants were administered the risky-gains task as previously described [81]–[83] and depicted in Figure 1. The task consists of 102 trials during which the numbers 20, 40, and 80 are presented in ascending order for one second each. Our choice of numbers, where each one is twice the preceding choice, was motivated by the observation that people typically reject gambles unless the amount that could be gained is at least twice the amount that could be lost [84]. The serial nature of the task, which required participants to make sequential fast judgments as to whether to accept or reject the amount displayed on the screen, was designed to capture the escalating tension that often accompanies naturalistic risky decision-making [17]. If the participant made a button press within one second of stimulus onset, that amount (20/40/80) could be added to their total winnings along with immediate visual and auditory feedback. When a 40 or 80 appear there is a chance that it appears in a different color with immediate feedback indicating the loss of 40- or 80-cents, respectively. When this happens the trial ends immediately and the participant may not make any more responses. Each of the 102 trials lasted 3.5s regardless of the participants’ choices and whether punishment was scheduled or not.

Participants were told that waiting for 40- or 80-cents was risky as, though it was possible to win more, it was also possible to lose that amount. Additionally, they were informed that though they would win less if they chose 20-cents, there was no risk of loss associated with this choice. That is, they could always win by choosing a 20-cents. However, participants were not told that there was no inherent advantage to choosing the risky (40, 80) choices over the safe choices (20). While the best response for each trial depended on whether there was a punishment scheduled for that trail or not, a strategy of selecting all 20 s would yield exactly the same winnings as selecting all 40 s or all 80 s.

Three different trial types were presented in a predetermined pseudo-randomized order: (1) non-punished (20, 40, or 80; n = 54), (2) punished 40 (n = 24), (3) punished 80 (n = 18) and six null trials. The relative number of punished and non-punished trials was chosen to guarantee that the strategy of consistently choosing 20-cents or always selecting 40- or 80-cents would yield the exact same winnings. Punishment occurred on a punished trial only if the participant failed to respond to the previous numbers on that trial (i.e. when holding out for 80 they did not respond to either the 20 or 40 stimulus). The relative frequency of safe (20) to risky (40 and 80) was used to quantify baseline risk-taking behavior. To investigate the sensitivity to punishment, defined here as the propensity to alter choice pattern on a trial immediately following a losing trial, the relative frequency of risky responses was examined as a function of the outcome of the previous trial, that is, punished versus non-punished risky trials.

This task has previously been used to assess risk-taking behavior in healthy volunteers [81]. This investigation revealed activation differences in insula, DLPFC, and posterior parietal cortex. Greater activation was observed in the insula during risky than during safe choices. The insula also showed significant activation on punished trials with activation magnitude predicting subsequent safe choices after a punished trial. In a purely behavioral study of stimulant users [82], we observed greater propensity for risk taking in the stimulant users but a similar degree of sensitivity to punishment in both the users and a control group. Those with higher measures of sensation seeking and impulsivity showed greater propensity to risk taking. Finally, Lee et al. [83] examined the effect of aging using the risky gains task. They reported greater levels of risk taking in younger people and more safe choices in older adults with faster reaction times to risky choices irrespective of group. They also observed greater insula and DLPFC activation for risky as compared to safe choices in the older adult group.

### MR Data Acquisition

Functional images were acquired in bottom-up interleaved axial slices using T2* weighted echo planar imaging (EPI). Images were acquired on two scanners: a 3T GE Discovery MR 750 (Milwaukee, WI) (252 volumes TR/TE = 2 s/30 ms, flip angle = 90°, 64×64 matrix, 40 axial slices, 3.75×3.75×3.0 mm voxels) and a 3T GE Signa HDx (Milwaukee, WI) (252 volumes, TR/TE = 2 s/30 ms, flip angle = 90°, 64×64 matrix, 40 3.0 mm (2.6 mm +0.3 mm gap) axial slices, 3.5×3.5 mm voxels). High-resolution T1-weighted fast spoiled gradient echo anatomical images (MR 750: TR/TE = 8.1 ms/3.17 ms, flip angle = 8°, 256×256 matrix, 172 sagittal slices, 1×1×1 mm voxels; Signa HDx: TR/TE = 7.77 ms/2.97 ms, flip angle = 8°, 256×256 matrix, 172 sagittal slices, 0.97×0.97×1 mm voxels) were acquired to permit subsequent activation localization and spatial normalization. Gradient echo field-maps were also acquired to permit compensation for geometric distortions caused by magnetic field inhomogeneity (MR 750: TR = 1 s, TE = 3.7/5.5 ms, flip angle = 60°, 64×64 matrix, 160 axial slices, 3.75×3.75×3 mm voxels; Signa HDx: TR = 1 s, TE = 3.5/5.5 ms, flip angle = 60°, 64×64 matrix, 160 3.0 mm (2.6 mm +0.3 mm gap) axial slices, 3.5×3.5 mm voxels). Stimuli were projected onto a screen at the participants’ feet and viewed with the aid of a mirror attached to the head coil.

### MR Data Analysis

Analyses were conducted using AFNI [85] and FSL [86]. T1-weighted images were skull-stripped using 3dSkullStrip and transformed to MNI152 standard space using FLIRT [87], [88] followed by nonlinear registration using FNIRT [89], [90]. Functional images were processed using FUGUE to compensate for B_0_ inhomogeneity [91]. Time series were motion corrected using an iterated linearized weighted least squares algorithm and aligned to the anatomical using a local Pearson correlation method [92] before being subjected to global mean-based intensity normalization. These were resampled to 3 mm isotropic voxels and transformed to standard space using the warp fields derived from transforming the T1 anatomy to MNI152 space. Finally, the time series were spatially blurred with a 4.2 mm isotropic FWHM Gaussian filter kernel within a mask derived from the T1 anatomy. Data were visually inspected to assess the quality of the warping and alignment. Preprocessed time series were subjected to multiple linear regression. Time series of interest, derived from the behavioral data (described below), were convolved with a gamma variate function [93] and subsequently normalized to a peak amplitude of 1.

Decision phase regressors were created such that they started at trial onset and ended either when the participant responded or was punished. Five regressors were defined: (1) 20 (safe), (2) win 40, (3) win 80, (4) lose 40, and (5) lose 80. General linear tests (GLT) of the safe (20) versus the risky win (40, 80) choices were computed for each participant. The baseline comprised all other time-points not accounted for by the regressors of interest. Additionally, six nuisance motion-related regressors (three translational and three rotational) and a 3^rd^ order Lagrange polynomial, which accounted for slow signal drift, were included in the baseline. Brain activation was operationally defined as percent signal change relative to baseline.

#### Task Related Group Analyses

The within-participant general linear tests of safe versus risky choices were subjected to linear mixed effects (LME) analyses in the R statistical analysis package [94]. As some participants had a lifetime diagnosis of major depressive disorder (MDD), a within-participant dichotomous variable indicating the presence/absence of MDD was also included in the model. Significant voxels were required to pass a voxel-wise statistical threshold (F_(1, 37)_ = 4.11, p = 0.05, uncorrected) and, to control for multiple comparisons, were required to be part of a cluster of no less than 1685 µL. The volume threshold was determined by a Monte-Carlo simulation that together with the voxel-wise threshold resulted in a 5% probability of a cluster surviving due to chance. The average percent signal change was extracted from the clusters so formed and a series of post hoc t-tests were conducted in R to examine the group and task interaction effects.

Since the HIV+ group consisted of more nicotine users than the HIV- group (see below), we conducted an additional analysis to investigate whether there was an effect of nicotine usage (FTND total score) on differential brain activation to risky versus safe choices. This was performed within the HIV+ group and accomplished using linear mixed effects models on the average contrast of risky versus safe choices in each of the clusters identified by the task related group analysis. In this analysis participant was treated as a random effect.

#### Controlling for Scanner Effects

Several recent studies have examined the effect of including MR data from multiple sites within the same analysis. Overall, these studies reported that inter-participant variance was anywhere from 7–44 times greater than that generated by site variance [95]–[98], even when group membership was confounded by site [99]. This suggests that scanner-induced variance is less likely to contribute to task or group-related effects. The inclusion of data from two scanners in our study effectively makes it a multi-site study and though the inclusion of site as a fixed-effect in the model examining group differences has been recommended [100], our study data was acquired in such a way that each participant was scanned only once on one scanner. It was therefore not possible to separately estimate the effects due to scanner and participant. Since scanner and participant are confounded in our study, we opted to include a dichotomous variable for scanner as the random effect in the linear mixed effects model described above.

#### Neuropsychiatric and Neuromedical Measures and Task-Related Brain Activations

Whole-brain voxel-wise Huber robust regressions [101], [102] were conducted in R [94] to examine the relationships between the neuromedical and neuropsychiatric measures and GLTs of safe versus risky win trials in the HIV+ group. We opted to perform regressions as some of the neuropsychiatric variables were confounded by diagnosis, and the neuromedical variable existed solely within the HIV+ group, precluding inclusion in the LMEs. Regressions were performed for the non-sexual sensation seeking and sexual compulsivity subscales of the KS4 based on our observation of between group differences on these measures (see below). A further regression examining the effect of nadir CD4 count was also conducted. Dichotomous variables for lifetime MDD diagnosis and scanner (for reasons outlined above) were included in these models. In the case of the nadir CD4 count, age and estimated duration of infection were also included in the model, as older participants or those with longer durations of HIV infection might have had lower nadirs and accumulated more damage due to HIV infection [103] that may manifest in altered brain functioning.

In all cases, regression coefficients and their corresponding t-values were split according to whether they demonstrated a positive or negative relationship with the GLTs. Thereafter, significant voxels were required to pass a voxel-wise statistical threshold (t(18) = 2.10, p = 0.05, uncorrected) and, to control for multiple comparisons, were required to be part of a cluster of no less than 1685 µL which resulted in a 5% chance of a cluster surviving due to chance. The volume threshold was determined in the same manner as above.

#### Overlap between Group and Regression Regions of Interest

To investigate whether between-group differences could potentially be attributed to neuropsychiatric and neuromedical variables, we assessed whether the regions identified from the task related analysis overlapped with those regions identified by the robust regression analysis. To accomplish this we computed the intersection of all those regions from the task related analysis with those from the regression analyses conducted solely within the HIV+ group. Since both of the maps included in this analysis included significantly different clusters, the resultant overlap maps can, therefore, also be regarded as statistically significantly different [104].

### Demographic and Clinical Scales Analysis

All analyses were conducted in R [94]. Between-group differences for demographics and clinical scales were assessed by means of Welch T tests for age, years of education, WRAT-4, BDI-II, POMS, KS4, BIS-11, FTND, speeded information processing, verbal fluency, learning, working memory, executive functions, and motor skills. Effect sizes were computed using Hedge’s g [105]. A linear mixed effects analysis (where participant was treated as a random effect) was conducted to investigate whether there was an effect on the BDI-II score of group, visit (baseline, scanning day), and their interaction. Group differences in GDS and days between the initial visit and scanning were assessed using Wilcox rank sum test. Effect sizes for these two measures were computed using the probability of superiority [106]. Group differences in gender, ethnicity, handedness, sexual orientation, the number of participants per group, the number of participants per scanner, and the number of individuals with a positive urine toxicology test for MJ were assessed using χ^2^ test of equal proportions. Using Spearman’s rank correlation test, we tested for the presence of a relationship between BDI-II score and the mean percentage signal change from each of the clusters resulting from the task-based whole brain analysis. This correlation was performed both solely within participants with lifetime diagnosis of MDD and within the sample as a whole.

### Task Analysis

The behavioral data gathered during task performance was subjected to a 3-way ANOVA in R [94]. In this model the effect of response choice (2 levels: safe (20) and risky (40, 80)), group (2 levels: HIV- and HIV+), punishment (2 levels: non-punished and punished trial), and their interactions were examined. This permitted assessment of the effects of choice behavior and susceptibility to prior punishment and how these varied by group.

## Results

### Demographics

There were no significant differences between the serostatus groups in age (t(37.99) = −0.74, p>0.1), handedness (*χ* ^2^(1) = 0.18, p>0.1), gender (*χ* ^2^(1) = 1.00, p>0.1), years of education (t(37.97) = −0.85, p>0.1), or ethnicity (*χ* ^2^(3) = 3.85, p>0.1). The groups differed in terms of sexual orientation, with the HIV+ group composed primarily of men who have sex with men, whereas the HIV- group was predominantly heterosexual (*χ* ^2^(1) = 13.81, p<0.001). Three participants tested positive for MJ (2 HIV+, 1 HIV-), a proportion that did not differ between groups (*χ* ^2^(1)≈0, p≈1). None of these participants met DSM-IV criteria for current or lifetime substance abuse or dependence. No participants tested positive for alcohol.

### Clinical Scales

There was no difference between the groups on GDS (W = 151.50, p>0.05). Three HIV- and seven HIV+ were classed as impaired (GDS >0.5); the proportions did not differ between the two groups (*χ* ^2^(1) = 0.84, p>0.1). There were no between-group differences observed in any of the speeded information processing, verbal fluency, learning, working memory, executive functions, and motor skills domains (all p≥0.1). There was a trend for the HIV- group to exhibit a higher WRAT-4 score than the HIV+ group (t(35.81) = 1.85, p = 0.07, g = 0.60). On the subscales of the KS4, the groups did not differ on the sexual sensation seeking subscale (t(34.04) = −1.79, p>0.05, g = −0.60) but the HIV-positives scored higher on sexual compulsivity (t(26.01) = −2.33, p<0.05, g = −0.70) and lower on non-sexual sensation seeking (t(36.32) = 2.02, p<0.05, g = 0.60). The groups showed no differences on the BIS-11 or any of its subscales (all p>0.05) (See Table 1.) Three HIV- and 11 HIV+ participants had a lifetime diagnosis of MDD (*χ* ^2^(1) = 4.37, p<0.05). The HIV+ group was comprised of marginally more nicotine dependent individuals than the HIV- group (*χ* ^2^(1) = 3.22, p = 0.07). There were no significant between-group differences on nicotine usage measured by the FTND (t(38) = −0.22, p>0.1). There were no significant differences between the two groups on other substance use characteristics (see Table 1). Significant between-group differences were observed on the BDI-II scale with the HIV+ group endorsing significantly greater levels of depression than the HIV- group both at the initial visit (t(22.53) = −4.07, p<0.001, g = −1.3) and at the time of scanning (t(25.71) = −3.16, p<0.01, g = −1.00). When determining the stability of BDI-II score over time, the HIV+ group had significantly higher BDI-II scores than the HIV- group (F_(1, 38)_ = 14.06, p<0.001), there was no effect of visit (F_(1, 38)_ = 1.81, p = 0.18), or an interaction between group and visit (F_(1, 38)_ = 1.79, p = 0.18). This suggests that the BDI-II score was stable over time. Finally, no significant between-group differences on the POMS were observed (t(29.02) = −1.75, p>0.05).

### Behavioral Task Results

A significant main effect of risk was evident (F_(1, 214)_ = 337.92, p<0.0001) with safe (M = 0.65) responses more likely than risky (M = 0.16). No significant effects of punishment (F_(1, 214)_ = 0.05, p>0.05, punished: M = 0.33, non-punished: M = 0.33) or group (F_(1, 214)_ = 0.21, p>0.05; HIV-: M = 0.33, HIV+: M = 0.32) were observed. No significant interactions of punishment × group (F_(1,214)_ = 0.06, p>0.05) or risk × punishment × group (F_(1,215)_ = 0.00, p>0.05) were observed. A marginally significant effect for risk × group (F_(1, 214)_ = 3.09, p = 0.08) was observed with the HIV- group selecting risky choices marginally more than the HIV+ group (HIV-: risky M = 0.19, safe M = 0.62; HIV+: risky M = 0.14, safe = 0.68). Consistent with our prior observations on this task [81], [82], a significant interaction of risk × punishment (F_(1,214)_ = 11.42, p<0.001) was observed wherein participants were more likely to chose the safe option when the prior trial was punished. See Table S2 for a complete list of the cell and marginal means.

### fMRI Task Results

#### Task Related Brain Activation

**Figure 2:**
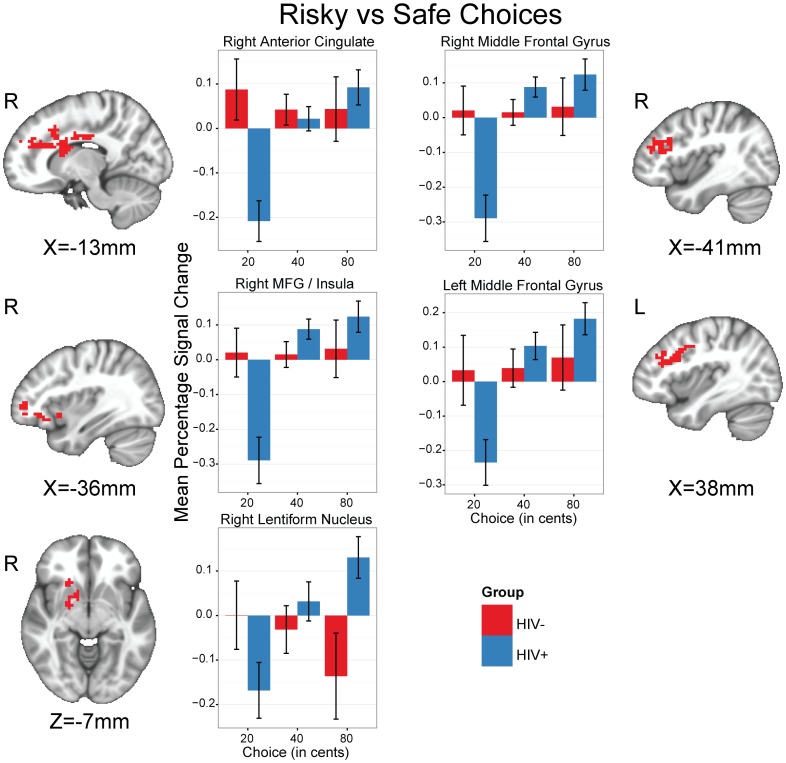
Brain regions identified by the between-group whole-brain analysis of the general linear test of risky (40, 80) versus safe (20) choices. The bar charts indicate percentage signal change and are the breakdown of responses within group and by choice (20, 40, 80) in these regions. Error bars indicate the standard error of the mean. L, left; R, right.

We identified eight regions where the HIV- and HIV+ groups differed (Table 2 and Figure 2). Cortical regions were located in the right anterior cingulate gyrus, inferior parietal lobule, superior frontal gyrus, and bilaterally in the middle frontal gyri. Two subcortical regions, one in each hemisphere with centers of mass in the left lentiform nucleus and right claustrum were identified. The left cluster extended dorsally from the ventral striatum to include portions of the head and body of the caudate and further extended to include parts of the putamen and anterior insula. The right cluster predominantly included portions of the head of the caudate and extended laterally to include the anterior insula. An additional subcortical cluster in the left thalamus was identified. Post hoc analyses were conducted to identify the directionality of these effects. Within the HIV+ group, activation was greater for risky relative to safe choices in the right ACC (t(31.31) = 4.56, p<0.001), left middle frontal gyrus (t(28.43) = 4.97, p<0.001), right middle frontal gyrus (t(27.86) = 6.39, p<0.001), left thalamus t(36.07) = 3.67, p<0.001), right claustrum (t(26.66) = 4.58, p<0001), and right superior frontal gyrus (t(28.76) = 3.21, p<0.001. Within the HIV- group, there were no significant differences between risky and safe choices (all p>0.1). For risky choices, the HIV+ group displayed greater activation than the HIV- group in left thalamus (t(70.83) = 2.66, p<0.01), left lentiform nucleus (t(68.71) = 3.41, p<0.01), and right claustrum (t(57.64) = 2.37, p<0.05). For safe choices, the HIV+ group displayed less activation than the HIV- group in the right ACC (t(32.45) = −4.1, p<0.001), left middle frontal gyrus (t(30.58) = −2.75, p<0.01), and right middle frontal gyrus (t(36.48) = −3.23, p<0.01). Within the participants with a lifetime diagnosis of MDD, none of these clusters showed a relationship with the BDI-II scores (all p>0.05). Across the sample as a whole, none of the clusters showed a relationship with the BDI-II scores (all p>0.05). Within the HIV+ group, there was no relationship between nicotine usage (FTND total score) and the contrast of risky versus safe choices in any of the aforementioned brain regions (all p>0.1).

**Table 2:** Functional brain regions identified as showing risk-related differences between the HIV- and HIV+ groups while they performed the risky gains task.

Structure	Hemisphere	BA	Volume	Center of Mass			Average
			(µL)	X	Y	Z	F-value^a^
Anterior Cingulate	R	24/32	21,249	−5	−16	27	7.16
Middle Frontal Gyrus	L	9	11,043	38	−15	33	6.92
Middle Frontal Gyrus	R	9/46	9,450	−41	−20	29	6.52
Thalamus	L		3,510	10	30	1	6.45
Lentiform Nucleus	L	13	3,348	20	−12	1	6.22
Claustrum	R		2,376	−26	−19	6	5.98
Inferior Parietal Lobule	L	40	2,349	41	65	44	5.42
Superior Frontal Gyrus	R	10	1,917	−26	−54	5	7.00

Center-of-mass coordinates, in radiological convention, are in the MNI152 standard space and structure labels are from the Talairach & Tournoux atlas [150]. BA, Brodmann Area; L, Left; R, Right. ^a^ F_(1, 36)._

#### Neuromedical and Neuropsychiatric Measures and Task-Related Brain Activations

Significant associations, detailed in Table S1, between differential brain responses to risky versus safe choice were identified for nadir CD4 and the Kalichman sexual compulsivity subscale. Those individuals with a higher CD4 nadirs exhibited lower activation in several regions including anterior cingulate gyrus, bilateral inferior parietal lobules, and middle frontal gyrus. Moreover, those individuals with higher ratings on the sexual compulsivity subscale of the KS4 showed lower activation in cingulate gyrus, and medial and middle frontal gyri. One region in the right pyramis was negatively associated with the non-sexual sensation seeking subscale of the KS4.

#### Overlap between Group and Regression Regions of Interest

**Figure 3:**
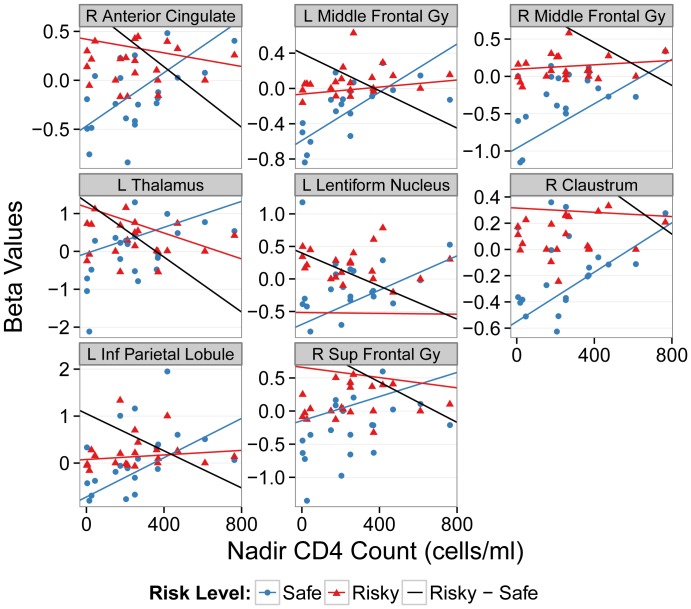
Within the HIV+ group, graphs of the relationship between nadir CD4 count and a subset of the voxels in each of the clusters depicted in Figure 2 and Table 2. The subset of voxels is the overlap between the clusters showing between-group task related functional differences and those clusters identified by the robust regression analysis conducted only within the HIV+ group. The black line is the robust regression line on the difference between risky and safe responses. The red and blue lines are robust regression lines of the safe and risky components of the black line. L, left; R, right; Inf, Inferior; Sup, Superior; Gy, Gyrus.

Several brain regions identified as showing task-related between-group differences overlapped with the regions identified in the regression analysis results just described. Regions that demonstrated a negative association with nadir CD4 overlapped with the between-group results in the right anterior cingulate, bilateral middle frontal gyri, left inferior parietal lobule, and right superior frontal gyrus. When the beta values for the risky and safe choices that contributed to this negative relationship were separated out, the difference between the two choice types appeared to be driven almost entirely by an increased response to the safe choice with greater nadir CD4 count (Figure 3).

**Figure 4:**
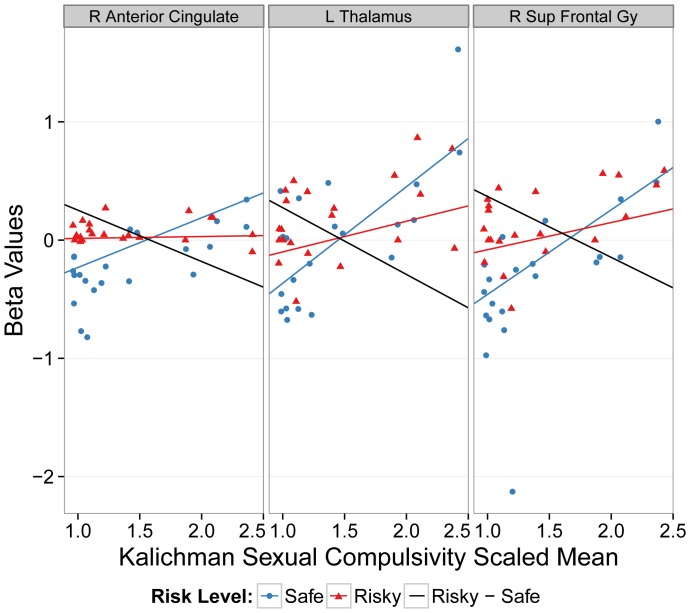
Within the HIV+ group, graphs of the relationship between Kalichman Sexual Compulsivity Scaled Mean and a subset of the voxels in three of the clusters depicted in Figure 2 and Table 2. The subset of voxels is the overlap between the clusters showing between-group task related functional differences and those clusters identified by the robust regression analysis conducted only within the HIV+ group. The black line is the robust regression line on the difference between risky and safe responses. The red and blue lines are robust regression lines of the safe and risky components of the black line. L, left; R, right; Sup, Superior; Gy, Gyrus.

Regions showing a negative relationship with the sexual compulsivity subscale of the KS4 overlapped with the task related clusters in the right anterior cingulate gyrus, left thalamus, and right superior frontal gyrus. Similarly, when the risky and safe beta values were separated out, the negative relationship appeared to be largely driven by the relationship between safe beta values and the compulsivity measure (Figure 4). No overlap between the non-sexual sensation seeking subscale of the KS4 and the between-group differences was observed.

## Discussion

Risky decision-making is a common feature of HIV-associated neurocognitive disorders, but its neural substrates within persons living with HIV are poorly understood. Here, we examined risky choice behavior in HIV+ individuals compared to seronegative individuals using functional magnetic resonance imaging. We observed significant between-group functional activation differences in a number of regions (ACC, DLPFC, caudate, and insula) critical to risk and reward processing despite broadly similar task performance between the two groups. In the overlap between the task-related regions of interest and those resulting from the robust regression analysis, those HIV+ individuals with greater sexual compulsivity measured by the KS4 and higher nadir CD4 count displayed lower differential responses to safe versus risky choices in many of the regions that showed between-group task related differences. Taken together, these results support the hypothesis that HIV alters risk-related processing in the basal ganglia, among other structures.

We observed significant between-group differences in left and right hemisphere clusters that included subcortical regions and small insular components. The subcortical constituents of this cluster included the ventral and dorsal striatum. These regions have been heavily implicated in reward related processing [47]. In non-human primates, single unit recordings have revealed populations of neurons in the caudate and putamen that fire in proportion to the value of an action irrespective of whether the action was subsequently executed [107], [108]. In humans, fMRI studies have shown that the ventral striatum is important to judging reward value [109], [110] and reduced striatal volume has been reported in HIV infection [111]. Similarly, increased blood oxygenation level dependent (BOLD) responses have been recorded in the dorsal striatum in response to anticipation of both primary (e.g., food) [112] and secondary (e.g., money) [109] rewards. Additionally, BOLD responses in the dorsal striatum have been shown to predict expected value of actual choices in a risky context [113]. This has lead some to suggest that the striatum is primarily involved in the prediction of reward value and that other brain regions (e.g., the insula) may be more important to quantifying risk [81], [114], [115]. The insula is thought to be important to integrating autonomic, visceral, and hedonic information [48], and it has been suggested that it is a critical neural substrate for selecting between internally and externally available homeostatically relevant information that serves to guide behavior [48]. Indeed, greater activation levels within the insula have be associated with risky choices in the task deployed here [81], [83]. Our results suggest that the differences in these clusters are predominantly driven by the HIV+ group who display progressively greater responses to the 20-, 40- and 80-cent choices compared to the HIV- group. Larger striatal activation for high gain/high risk trials has been observed in a study of decision making under risk [116]. Our observation of increased activation with greater value of the potential gain may be significant insofar as it may be the functional neuroanatomical realization of over-valuation of the potential benefit of risky choices which has previously been reported in HIV+ individuals [19]. Alternatively, it is possible that increasing activation to progressively higher valued risky choices in these subcortical clusters may be related to damage caused to the basal ganglia by HIV [2] and may thus be an adaptive functional response to this injury [25]. Disambiguation of these alternatives will require future studies.

There were significant between-group differences in activation level in the right anterior cingulate between the HIV+ and HIV- groups. This dorsal region of the ACC is thought to be important to cognitive processes [117], reward-based learning and affective valence [118], [119]. Indeed, prior studies have indicated that the ACC is critical to judging the magnitude and likelihood of risky outcomes [120], [121], and others have suggested that ACC activation may be related to encoding of action cost [122] and action selection for uncertain rewards [123]. It has also been suggested that the ACC may perform a cost benefit analysis to guide action selection [124]. Activation of the ACC in a risky decision making paradigm has been associated with risk-aversive behavior, whereas deactivation was correlated with risk-seeking behavior [125]. Finally, the DACC has been proposed to play a critical role in the detection of response conflict [55]. In the context of decision making, tension between reward seeking and loss avoidance may naturally give rise to a state of conflict [126]. The greater DACC activation for the risky choices in the HIV+ group may indicate that they are more sensitive to conflict between risk seeking and loss avoidance behavior or, alternatively, the cost of losing. Here, it may be the case for the HIV+ group that the possibility of losing on the risky options outweighs that benefit of winning. The opposite may be the case for the HIV- group: the cost of losing on the risky choices may not loom large and be reflected in the lower activation for the risky choices. This stands in contrast to the observations on the IGT where HIV+ individual make more selections from disadvantageous decks [19], [20]. However, recent evidence suggests that this effect may be more common in individuals with HAND [21]. The small number of participants (n = 7) with HAND in the present study precluded examination of this possibility here. Therefore, further studies with larger numbers of participants with and without HAND are required to assess whether the present patterns of activation would vary by diagnosis.

Finally, consistent with prior finding of this task in [83], a between-group activation emerged bilaterally in the middle frontal gyrus, a component of the DLPFC. DLPFC is thought to be one of the seats of higher executive brain function [127], and it has been suggested that DLPFC plays a key role in the maintenance of goal-directed behavior necessary for successful task performance when alerted to the presence of conflicting behavioral choices by the DACC [55]. Several studies have examined the role played by the DLPFC during risky decision-making. In an fMRI study, activation of the DLPFC has been identified in decision making under uncertainty [126]. Using repetitive transcranial magnetic stimulation, a technique that can transiently suppress neuronal function [128], it has been shown that interrupting activity in the right DLPFC can increase risk-taking behavior [57], [58]. Another technique, transcranial direct current stimulation, has also been employed to assess the role of DPLFC in risky decision-making. In this method, low-voltage direct current is passed through the brain using electrodes placed on the scalp [129]. Using this technique, it has been shown that increasing excitability of the right or left DLPFC leads to risk-aversion and suggests that DLPFC may be critical to the suppression of riskier choices [56].

DACC and DLPFC are extensively interconnected [59] and are components of a dorsal executive circuit that is critical to performance monitoring [49]–[53] and maintenance of goal-directed behavior [54], [55]. Furthermore, this dorsal circuit interacts with the ventral circuit (consisting of insular and striatal regions) to predict stimulus-reward value and guide future behavior. The striatal regions of this circuit are known to be injured by the HIV virus [2], and documented cognitive deficits in HIV infection are consistent with fronto-striatal white matter damage that has been attributed to HIV infection [34]. Our observation of increased DACC and DLPFC activation in the HIV+ group in the presence of broadly similar task performance may therefore be an adaptive functional response [25], [30] wherein additional cortical resources are recruited to maintain task goals [130]. This may be necessitated by aberrant information provided by other cortical and subcortical structures to which the dorsal circuit is connected and that may have been damaged by HIV infection. Indeed, additional functional recruitment in the presence of equivalent task performance has previously been observed in HIV+ individuals performing visual attention tasks [26], [27], [31], [32], working memory tasks [25], [26], and a simple finger tapping task [30]. This has led some to suggest that functional brain differences, in the absence of behavioral changes, may precede clinical signs of cognitive impairment [25], [38].

Since, relative to the seronegative group, the HIV+ group displayed elevated sexual compulsivity – a factor that has been associated with risk of HIV infection [131] – we investigated whether sexual compulsivity would show any relationship between differential activation to safe versus risky choices in the HIV+ group. Of those regions-of-interest (ROIs) identified as showing between-group task related differences, a subset of voxels in three of those ROIs (see Table 3) also showed a relationship with sexual compulsivity. We separated out the risky and safe components of this relationship. The change in differential responses to risky versus safe responses appeared to be driven by an overall increase in activation to both risky and safe choices with increasing compulsivity (see Figure 4). While there are, to our knowledge, currently no brain imaging studies that examine the relationship between functional activation and sexual compulsivity, neurobiological models of obsessive-compulsive disorder, however, have implicated excessive activity in fronto-striatal circuits, particularly in orbitofrontal, ACC, thalamus and caudate [132]–[136]. This suggests that those HIV+ individuals with elevated sexual compulsivity may be characterized by on overall increase of activation in fronto-striatal regions.

**Table 3:** Overlap between regions showing task related between-group differences (from Table 2) and regions identified by robust regression relating brain activity to neuromedical and neuropsychiatric measures.

Structure	Hemisphere	BA	Volume	Average	Average
			(µL)	*t*-value^a^	β value
**Term: Nadir CD4 Polarity: Negative**					
Anterior Cingulate	R	24/32	1,215	−2.37	−0.0011
Middle Frontal Gyrus	L	9	216	−2.89	−0.0009
Middle Frontal Gyrus	R	9/46	621	−2.57	−0.0011
Thalamus	L		756	−2.71	−0.0024
Lentiform Nucleus	L	13	108	−2.84	−0.0012
Claustrum	R		378	−1.52	−0.0006
Inferior Parietal Lobule	L	40	594	−2.21	−0.0013
Superior Frontal Gyrus	R	10	486	−2.39	−0.0011
**Term: Kalichman Sexual Compulsivity Scale Polarity: Negative**					
Anterior Cingulate	R	24/32	3,456	−2.61	−0.42
Thalamus	L		81	−2.42	−0.61
Superior Frontal Gyrus	R	10	783	−2.64	−0.51

Term refers to the neuropsychiatric or neuromedical explanatory variable in the regression model from which the clusters were derived. Polarity refers to the sign (positive or negative) of the regression coefficients from which the cluster was generated. BA, Brodmann Area; L, Left; R, Right. ^a^ t_(17)._

Within the HIV+ group, we also investigated whether nadir CD4 counts would show any relationship to differential activation to safe versus risky choices. We observed that subsets of the voxels identified as showing between-group task related differences also showed a relationship to nadir CD4 count. As depicted in Table 3 and Figure 3, greater nadir CD4 counts were negatively associated with decreased differences in response to risky versus safe choices in all of the task related ROIs. When the risky and safe components of this difference were separated out, the disparity between risky and safe responses appeared to be driven by increased activation to safe responses with greater nadir CD4 count. This suggests that differential activity to safe versus risky choices may be, in part, predicted by nadir CD4 count. Furthermore, it may be the case that those individuals with higher nadir CD4 counts may have activations patterns more similar to that of seronegative individuals than those with lower CD4 nadirs. This finding is consistent with the so-called “legacy events” hypothesis wherein historical immune-compromise increases the vulnerability of HIV-associated central nervous system injury [30], [36], [61], [65], [103].

This study has several limitations. The data were acquired from the two groups of participants on two different scanners. Though we attempted to minimize the differences between the protocols on both scanners and to account for this source of variation in our models, future studies are required to replicate the results reported here in the absence of this potential confound. This is a cross-sectional study, and thus we cannot address whether the differences reported here arose as a consequence of HIV infection or whether they predated HIV infection. Future longitudinal studies are required to determine whether factors such as duration of infection or the use of anti-retroviral therapy may influence impairment. In light of the recent report that risky decision-making is more prevalent in individuals with HAND [21], future studies should examine whether the effects reported herein are driven by more impaired individuals. HIV infection has been associated with risky decision making in individuals at risk for [18] and infected with HIV [19], [20]. Our observation in the behavioral analysis of a marginally significant interaction of risk and group with HIV- participants, counter-intuitively, choosing more risky options than the HIV+ group therefore warrants further investigation in a larger sample where the source of this observation may be more fully explored. Recent studies have reported differences in brain structure and function between homosexual and heterosexual men [137], [138]. The confounding of the serostatus groups by sexual orientation in the present sample prevented us from investigating whether HIV-infection status interacts with sexual orientation in risky decision-making. Future studies with a larger non-confounded sample are required to elucidate this issue. Depression is common in many HIV+ individuals [73] and inclusion of such individuals arguably makes the present sample more representative of the individuals seen in clinics and thus improves the generalizability of our results. Nevertheless, future studies should be conducted in groups with equivalent levels of depression to determine the specificity of the results reported herein. Risk for HIV infection has been associated with substance use (cf. [139]–[142]) and, as with depression, our inclusion of participants with histories of such behaviors arguably makes our sample more representative of the HIV-infected population. Nevertheless, substance use has been independently associated with functional brain changes in many of the brain regions reported here (e.g., [82], [143]–[146]). Investigating the specificity of the changes reported here in larger samples of HIV+ individuals with and without a history of substance use is therefore crucial. Given the preliminary evidence which suggests that anti-retroviral therapy (ART) can effect recovery of brain function to patterns typical of healthy controls [147], it remains unclear whether ART can influence risky choice behavior or the brain processes underlying it. Since our study was underpowered to examine this question, future studies with larger cohorts are required to examine this issue. Our sample of participants is almost exclusively male, limiting generalizability to the female population. Future studies with a larger sample of females are required to address this issue. Finally, our study concentrated on the time period prior to choosing between risky and safe options, future studies should investigate whether HIV+ individuals, compared to seronegative individuals, exhibit differences in sensitivity to the outcomes of these choices.

## Conclusions

In summary, the present study examined the functional neuroanatomy of risky decision-making in HIV+ individuals compared to seronegetative individuals. The HIV+ group displayed altered functional responses to safe and risky choices in several brain regions compared to the seronegetative group. Specifically, these regions included portions of the anterior cingulate, ventral and dorsal striatum, insula, and bilateral DLPFC. These results are consistent with and further support the role of these structures in risky decision-making [47], [48], [56]–[58], [148], [149]. We observed greater DACC and DLPFC activation to risky choices in the HIV+ group in the presence of broadly similar task performance between the two serostatus groups. This suggests an adaptive functional response [25], wherein additional cortical resources are recruited to maintain task goals [130]. This may be in response to aberrant information provided by other cortical and subcortical structures to which these regions are connected and that may have been damaged by HIV infection. Within the HIV+ group, we observed increased activation in the right ACC, left thalamus, and right superior frontal gyrus as a function of increased sexual compulsivity. This suggests that those HIV+ individuals with elevated sexual compulsivity may be characterized by on overall increase of activation in fronto-striatal regions. Finally, we also observed that greater nadir CD4 count was significantly associated with greater activation to safe choices rather than risky options in all of the regions displaying between-group task-related differences. This suggests that HIV infection may alter risk-related neural processing.

## Supporting Information

Table S1 Regions showing differences between differential brain responses to risky versus safe choices and neuropsychiatric measures.
(XLSX)

Table S2 Marginal and cell means from the ANOVA for the behavioral analysis.
(XLSX)
